# Mannequin or standardized patient: participants' assessment of two training modalities in trauma team simulation

**DOI:** 10.1186/1757-7241-17-59

**Published:** 2009-11-25

**Authors:** Torben Wisborg, Guttorm Brattebø, Åse Brinchmann-Hansen, Kari Schrøder Hansen

**Affiliations:** 1 The BEST Foundation: Better & systematic trauma care, Hammerfest Hospital, Department of Acute care, Hammerfest, Norway; 2 Dept. of Anaesthesia & Intensive Care, Haukeland University Hospital, Bergen Norway; 3 Norwegian Medical Association, Oslo, Norway; 4 Longyearbyen Hospital, Norway

## Abstract

**Background:**

Trauma team training using simulation has become an educational compensation for a low number of severe trauma patients in 49 of Norway's 50 trauma hospitals for the last 12 years. The hospitals' own simple mannequins have been employed, to enable training without being dependent on expensive and advanced simulators. We wanted to assess the participants' assessment of using a standardized patient instead of a mannequin.

**Methods:**

Trauma teams in five hospitals were randomly exposed to a mannequin or a standardized patient in two consecutive simulations for each team. In each hospital two teams were trained, with opposite order of simulation modality. Anonymous, written questionnaires were answered by the participants immediately after each simulation. The teams were interviewed as a focus group after the last simulation, reflecting on the difference between the two simulation modalities. Outcome measures were the participants' assessment of their own perceived educational outcome and comparison of the models, in addition to analysis of the interviews.

**Results:**

Participants' assessed their educational outcome to be high, and unrelated to the order of appearance of patient model. There were no differences in assessment of realism and feeling of embarrassment. Focus groups revealed that the participants felt that the choice between educational modalities should be determined by the simulated case, with high interaction between team and patient being enhanced by a standardized patient.

**Conclusion:**

Participants' assessment of the outcome of team training seems independent of the simulation modality when the educational goal is training communication, co-operation and leadership within the team.

## 

The treatment of severely injured patients is challenging, and several studies have indicated that the emergency room phase poses a high risk for protocol deviations and substandard treatment[1,2]. The reported rate of avoidable death after injury is from 10 to 25 percent [3,4]. Training of trauma teams with simulated patients is an option when the regular case load is insufficient to maintain and develop experience and expertise in treating these patients. We have developed the BEST program (BEST: Better and systematic trauma care) during 12 years [5], with the intention to enable hospitals to arrange their own local training after an introductory course. For this purpose we used ordinary resuscitation mannequins as simulated patients. They were available in all 49 hospitals which have had one or more training courses, but are of low fidelity compared to advanced simulators and standardized live patients. Advanced simulators are expensive, difficult to move and demands experienced instructors and operators [6,7]. They do not fulfil the BEST programs' wish of enabling hospitals to arrange further local training on their own, without having to raise substantial funds for equipment. Standardized patients, given a thorough instruction, would be an interesting alternative because they are alive, realistically heavy and able to communicate, and may thus fit into the program's philosophy. Still, these standardized patients will have normal circulatory status, skin temperature and appearance, and cannot be intubated or subjected to other invasive procedures. The need for an experienced facilitator would thus not be reduced by this approach. There is little knowledge about the educational outcome of team training with different patient models, and we wanted to examine the participants' assessment of their educational outcome after training with either a standardized patient or a simple resuscitation mannequin.

## Materials and methods

Five hospitals received standard BEST trauma team courses consisting of three and a half hours of didactical lectures and discussion for all team members, focusing on algorithms for trauma care and team communication, leadership and co-operation in trauma teams [8]. Two complete trauma teams at each hospital, composed according to the hospitals' local protocols, were given two consecutive simulations with structured debriefing after each simulation. Training was done in the hospitals' ordinary trauma rooms. Each team had one simulation with a mannequin and one with a standardized patient (a carefully instructed nurse or medical student). The teams were randomly allocated to have the mannequin or the standardized patient as their "patient" in the first simulation, and vice versa in the next. The hospital and the participants were not informed about the presence of the standardized patient until immediately before simulation, and the BEST instructors were not aware of the team composition and order of appearance before simulation. The team members were informed that they could withdraw from the training if they felt so, and that the object of study was the team, not the individual. In all hospitals, this was the first BEST course ever. The participants answered an individual questionnaire after each simulation. Questionnaires were anonymous, and could not be referred to individuals. The respondents indicated their assessment of different aspects of the simulation on 100 mm visual analogue scales (VAS) where VAS 0 denoted "little" and 100 "high". The VAS was anchored with a few words describing each extreme, but otherwise without tick marks.

The participants were asked to assess the degree of realism ("To which extend do you think that it was difficult to imagine the mannequin/standardized patient as a real patient?") and the degree of personal embarrassment ("To which degree did you find it embarrassing to treat a mannequin/standardized patient instead of a real patient, in the way that the educational outcome was reduced?") after their first simulation experience.

All participants were asked to compare the two simulation modalities after their second simulation by posing the question; "In conclusion, how do you assess the educational outcome by using a mannequin as compared to a standardized patient?" and the VAS was anchored with 0 = prefer mannequin, 50 = equal and 100 = prefer standardized patient.

All teams progressed through the same two cases irrespective of the modality used. The first case was an unconscious pedestrian who had been hit by a car, and gradually awakened during the initial treatment; the second was an alert patient with severe internal haemorrhage after injury developing progressing signs of circulatory collapse according to the treatment given.

After each simulation session the team had a 30 minutes structured debriefing and discussion. Eventually, each team was interviewed after their second debriefing in a focus group format, asking for the participants' opinion on realism, credibility, intensity and embarrassment during the simulation situation [9,10]. Due to disturbing noise in the room audio recording was impossible, and the answers were written down during the session. The groups were moderated by one of the authors (ÅBH).

Data were analyzed with the SPSS 11.0. T-testing, and one-way analysis of variance (ANOVA) with Bonferroni's correction, was used for comparisons of means. Chi-square testing was used for comparing frequencies. A p-value of less than 0.05 was considered statistically significant. Means are given with standard deviation in brackets (SD). The focus group conversations were analyzed using a grounded theory approach [11,12].

## Results

The hospitals were all district general hospitals serving populations from 15,000 to 100,000. A total of 104 trauma team members participated in the simulations, of which 32 were doctors, 53 nurses and 19 radiographers, lab technicians etc. There were 51 participants in the teams that started with standardized patient and proceeded to a mannequin, while there were 53 in the opposite groups. There were no differences between the two groups concerning distribution of professions.

Individual assessments of the educational outcome after each simulation are summarized in Table 1. The five teams that started with a mannequin consistently assessed their educational outcome above the five teams starting with a standardized patient, independent of training modality.

**Table 1:** Participants' assessment of their educational outcome of each simulation session immediately after each session.

	Mannequin	Standardized patient	
Mannequin first (group 1)	80 (12)	79 (12)	n.s.
Standardized patient first (group 2)	71 (18)	70 (15)	n.s.

53 participants started with a mannequin (group 1) and 51 with a live standardized patient (group 2), n = 104. 100 mm VAS, mean with (SD). Participants that were exposed to mannequin first were subsequently exposed to standardized patient and vice versa.

The findings concerning assessed realism and embarrassment are given in Table 2.

**Table 2:** Participants' assessment of the degree of realism and their personal feeling of embarrassment when handling either the mannequin or the live standardized patient.

	Mannequin	Standardized patient	
Realism	53 (23)	53 (24)	n.s.
Embarrassment	31 (23)	27 (21)	n.s.

Answers given after the first simulation session. 53 participants started with mannequin (group 1) and 51 with standardized patient (group 2), n = 104. 100 mm VAS, mean with (SD).

The final individual comparison between mannequin and standardized patient revealed a VAS of 68 (SD 21) (0 = prefer mannequin, 50 = equal and 100 = prefer standardized patient). There was a significant difference between the two groups, the group exposed to standardized patient first answering 60 (SD 21) and the group exposed to mannequin first answering 76 (SD 17) p < 0.005. There were no significant differences between professions in their assessment of the two training modalities.

The focus group interviews revealed no differences in the dimension personal educational outcome. After two simulations the groups expressed no preferences as for mannequin or standardized patient. Concerning the question of realism the focus groups' discussions revealed that realism was related to the clinical condition being simulated. If the patient in the scenario was supposed to be conscious and active, especially the nurses said they preferred a live modality that could interfere in the treatment and also give important information. The simulation would then be more realistic and closer to a real clinical situation. However, in a situation with an unconscious patient it was as realistic to have a mannequin as a standardized patient. Many expressed that they felt freer to do various procedures on a mannequin compared to a standardized patient. Many participants said they were afraid that full engagement in the simulation could make them put needles into the standardized patient's arms or in other ways 'harm' the live model.

As to the question of credibility in this situation the focus groups discussions revealed no difference between using a standardized patient or a mannequin. The same results came from discussing intensity or involvement in the simulation situation. The teams did not experience any embarrassment as to using a standardized patient versus a mannequin.

In conclusion the focus group discussions revealed no special preference for either using a mannequin or a standardized patient in general, but a slight preference for using a standardized patient if the simulated patient is supposed to be able to talk and interact with the trauma team. If the patient was supposed to be unconscious, no preference was expressed.

## Discussion

This study shows that the training modality used for simulation during multiprofessional trauma team training seems to be of little influence to the perceived educational outcome. Interestingly, the participants evaluated the two different methods remarkably similar. There was a small, but significant difference between the five teams starting with the mannequin compared to the teams who started with the standardized patient, but within each group of teams we found no difference in evaluation of the perceived educational outcome. We thus consider this difference between the two groups coincidental. When considering realism of the training modality and the degree of embarrassment experienced when handling the model instead of a real patient, we found no differences. At the end of the day, however, the respondents all slightly favoured the standardized patient for the mannequin.

Simulators appeared in anaesthesia as one of the first places in medicine [13,14]. They have been used for many different purposes, from skills training to decision making, from individual to group training [15]. Much emphasis has been put on increasing the fidelity, but as the simulators become increasingly advanced their mobility decreases and the demand for experienced operators and instructors increases. So do inevitably the costs. There has been a tendency to view the more advanced simulator models as superior to simpler approaches, an assumption not based on current knowledge [16]. Some studies have tried to compare high- and low-fidelity simulators for the same educational goal [17-19]. The results seem to indicate that higher fidelity favours learning when practical skills are trained and assessed, although not unequivocal. However, when training multiprofessional teams with emphasis on communication, leadership and cooperation arranged locally at the trainee's workplace, we have found no comparable studies.

The conclusions of this study must be considered in the context where it was done. Our findings are not necessarily transferable to training settings where one aims at training in interaction with outspoken, verbal patients, or where input from patient physiology to monitors is considered important. Similarly, if the educational goal is to train specific skills requiring invasive procedures on the mannequin like endotracheal intubation or chest tube insertion, neither simple mannequins nor standardized patients are suitable. In addition, the possibilities for data acquisition were determined by the circumstances, and the focus group interviews thus had to be done under suboptimal conditions. However, we still consider the findings valid for the setting studied and with value for careful transfer to other settings with similar educational goals.

In conclusion, this study shows that there seems to be little difference between a simple resuscitation mannequin and a standardized patient when the educational goal is multiprofessional team training with emphasis on team communication, leadership and co-operation.

## Competing interests

The BEST Foundation is a non-profit network of the Norwegian public hospitals with responsibility for acute care of trauma victims. Full financial disclosure is practised at the Foundations website http://www.bestnet.no. None of the authors had any financial or other competing interest in the study or the publication of it.

## Authors' contributions

ÅBH, GB and TW did the data collection, analysis and the first draft writing. All authors participated in the analysis and final editing of the manuscript. TW conceived the study.

## References

[B1] Maio R Burney R Gregor M Baranski M A study of preventable trauma mortality in rural Michigan Journal of Trauma-Injury Infection & Critical Care 1996 41 1 83 90 10.1097/00005373-199607000-00013 8676428

[B2] Esposito T Sanddal N Hansen J Reynolds S Analysis of preventable trauma deaths and inappropriate trauma care in a rural state Journal of Trauma-Injury Infection & Critical Care 1995 39 5 955 962 10.1097/00005373-199511000-00022 7474014

[B3] Gorman D Teanby D Sinha M Wotherspoon J Boot D Molokhia A Preventable deaths among major trauma patients in Mersey Region, North Wales and the Isle of Man Injury 1996 27 3 189 192 10.1016/0020-1383(95)00200-6 8736294

[B4] Esposito T Sanddal T Reynolds S Sanddal N Effect of a voluntary trauma system on preventable death and inappropriate care in a rural state Journal of Trauma-Injury Infection & Critical Care 2003 54 4 663 669 discussion 669-670 10.1097/01.TA.0000058124.78958.6B 12707527

[B5] Wisborg T Brattebo G Brinchmann-Hansen A Uggen PE Hansen KS Effects of nationwide training of multiprofessional trauma teams in norwegian hospitals J Trauma 2008 64 6 1613 1618 10.1097/TA.0b013e31812eed68 18545132

[B6] Issenberg SB Gordon MS Gordon DL Safford RE Hart IR Simulation and new learning technologies Med Teach 2001 23 1 16 23 10.1080/01421590020007324 11260734

[B7] Holcomb J Dumire R Crommett J Stamateris C Fagert M Cleveland J Dorlac G Dorlac W Bonar J Hira K Aoki N Mattox KL Evaluation of trauma team performance using an advanced human patient simulator for resuscitation training Journal of Trauma-Injury Infection & Critical Care 2002 52 6 1078 1085 discussion 1085-1076 10.1097/00005373-200206000-00009 12045633

[B8] Wisborg T Brattebo G Brattebo J Brinchmann-Hansen Å Training Multiprofessional Trauma Teams in Norwegian Hospitals using Simple and Low Cost Local Simulations Education for Health 2006 19 1 85 95 10.1080/13576280500534768 16531305

[B9] Kitzinger J Qualitative Research: Introducing focus groups BMJ 1995 311 7000 299 302 7633241 10.1136/bmj.311.7000.299 PMC2550365

[B10] Malterud K Qualitative research: standards, challenges, and guidelines Lancet 2001 358 9280 483 488 10.1016/S0140-6736(01)05627-6 11513933

[B11] Hartman J Grounded Theory. Generating theory on an empirical basis. [Grundad teori. Teorigenerering på empirisk grund.] 2001 Lund, Sweden: Studentlitteratur

[B12] Glaser BG Strauss A The Discovery of Grounded Theory: Theories for Qualitative Research 1967 Mill Valley, CA: Sociology Press

[B13] Gaba D Howard S Flanagan B Smith B Fish K Botney R Assessment of clinical performance during simulated crises using both technical and behavioral ratings[see comment] Anesthesiology 1998 89 1 8 18 10.1097/00000542-199807000-00005 9667288

[B14] Small SD Wuerz RC Simon R Shapiro N Conn A Setnik G Demonstration of high-fidelity simulation team training for emergency medicine Acad Emerg Med 1999 6 4 312 323 10.1111/j.1553-2712.1999.tb00395.x 10230983

[B15] Issenberg SB McGaghie WC Petrusa ER Lee Gordon D Scalese RJ Features and uses of high-fidelity medical simulations that lead to effective learning: a BEME systematic review Med Teach 2005 27 1 10 28 10.1080/01421590500046924 16147767

[B16] Beaubien JM Baker DP The use of simulation for training teamwork skills in health care: how low can you go? Qual Saf Health Care 2004 13 Suppl 1 i51 56 10.1136/qshc.2004.009845 15465956 PMC1765794

[B17] Lee KH Grantham H Boyd R Comparison of high- and low-fidelity mannequins for clinical performance assessment Emerg Med Australas 2008 20 6 508 514 10.1111/j.1742-6723.2008.01137.x 19125830

[B18] Crofts JF Bartlett C Ellis D Hunt LP Fox R Draycott TJ Training for shoulder dystocia: a trial of simulation using low-fidelity and high-fidelity mannequins Obstet Gynecol 2006 108 6 1477 1485 17138783 10.1097/01.AOG.0000246801.45977.c8

[B19] Grady JL Kehrer RG Trusty CE Entin EB Entin EE Brunye TT Learning nursing procedures: the influence of simulator fidelity and student gender on teaching effectiveness J Nurs Educ 2008 47 9 403 408 10.3928/01484834-20080901-09 18792707

